# Editorial: Interplay of immunity and ECM in cancer

**DOI:** 10.3389/fonc.2025.1704061

**Published:** 2025-10-09

**Authors:** Albina Fejza, Federica Benvenuti, Eva Andreuzzi

**Affiliations:** ^1^ Deparment of Medical Biochemistry and Biotechnology, University, for Business and Technology (UBT), Prishtine, Albania; ^2^ Deparment of Cellular Immunology, International Centre for Genetic Engineering and Biotechnology, Trieste, Italy; ^3^ Deparment of Obstetrics and Gynecology, IRCCS Materno Infantile Burlo Garofolo, Trieste, Italy

**Keywords:** cancer, tumor microenvironment, extracellar matrix, immunotherapy, biomarkers

In recent decades, cancer research has increasingly focused on understanding tumor heterogeneity to facilitate molecular classification and design personalized therapies. In this view, a critical yet still underexplored element is the tumor microenvironment (TME), composed of stromal, vascular, and immune cells embedded in the extracellular matrix (ECM) (1). These components interact dynamically, shaping tumor development and progression (2). The ECM, in particular, influences tumor angiogenesis and immune cell infiltration and activation, thereby impacting on pivotal aspects as hypoxia, cancer cell dissemination, and the tumor immune microenvironment (3). The latter represents the target of immunotherapeutic agents, which have revolutionised the treatment of certain cancer types and provided an efficacious option for subgroups of patients in recent decades (4–6). Nevertheless, a considerable number of patients fail to respond or experience adverse effects, underscoring the necessity for a more profound comprehension of the mechanisms underlying these processes and the identification of biomarkers that can effectively predict therapeutic outcomes. The principal objective of this Research Topic is to provide a platform for the publication of novel research findings pertaining to the mechanisms that regulate the crosstalk between the extracellular matrix (ECM) and the immune system. In addition, it aims to showcase valuable biomarkers and novel therapeutic targets that will facilitate the development of more effective patient management strategies.

The recent advancements of immunotherapy have been promising for the treatment of cervical cancer (CC), however, the overall response rate suggested that the treatment is ineffective in subsets of patients. Gao et al. analyzed RNA-seq profiles from public datasets, identifying LAMA4 as a significantly overexpressed ECM-related gene (ERG) in CC compared to normal tissue. High LAMA4 expression correlated with poor overall and progression-free survival, as well as reduced immunotherapy response. Unexpectedly, LAMA4 was also detected in the nucleus, possibly due to ECM remodeling and nuclear translocation of fragments. Functionally, LAMA4 overexpression increases ECM rigidity, preventing T-cell infiltration, and activates integrin-mediated pro-survival signaling, fostering immune resistance. Thus, LAMA4 emerges as both a biomarker of poor prognosis and an active driver of an immunosuppressive microenvironment.

ECM stiffness, primarily mediated by collagen crosslinking, regulates immune cell trafficking and tumor invasiveness. Lysyl oxidases (LOX) are key enzymes in this process. Jiang et al. investigated LOXL4 in triple-negative breast cancer (TNBC) and found that it induces matrix metalloproteinase-9 (MMP-9) expression through NF-κB activation. LOXL4 promotes annexin A2/integrin-β1 accumulation on the cell surface, which triggers the TRAF4–TAK1–NF-κB signaling pathway, enhancing MMP-9 transcription and secretion. This mechanism increases TNBC cell invasiveness and positions LOXL4 as a potential therapeutic target for metastatic disease.

Gao et al. investigated neutrophil extracellular traps (NETs) in multiple myeloma (MM). Using Cox and LASSO regression, they identified 64 NET-related and differentially expressed genes, constructing a six-gene risk score (CTSG, HSPE1, LDHA, MPO, PINK1, VCAM1). This model stratified patients into high- and low-risk groups with significantly different survival outcomes, confirmed as an independent prognostic factor. High-risk patients displayed fewer immune cells, poorer clinical markers, and worse survival but greater sensitivity to drugs such as bortezomib. These findings suggest NET-related gene signatures as both prognostic markers and predictors of therapy response in MM.

This Research Topic also encompasses reviews that offer insights into the highly complex characteristics of the TME.

Mancini et al. reviewed the multifaceted roles of the ECM in cancer, emphasizing both its biomechanical and biochemical influences. They detailed how ECM stiffness and organization impact tumor progression, and how stromal and immune cells modulate ECM remodeling. The review highlighted ECM as not just a structural scaffold but a dynamic regulator of signaling pathways that drive cancer growth and therapy resistance.

Mei et al. explored hyperbaric oxygen therapy (HBOT) as a novel strategy to remodel the TME. HBOT increases tissue oxygenation, enhances mitochondrial activity, and generates reactive oxygen species that degrade collagen and fibronectin, softening the ECM. This facilitates immune and therapeutic cell penetration. HBOT also activates T and NK cells, stimulates matrix-degrading enzymes, reduces regulatory T cells, and suppresses HIF-1α, collectively promoting ECM breakdown and anti-tumor immunity. Their review suggests HBOT as a promising adjunct to conventional cancer therapies.

Matrix metalloproteinases (MMPs) are central to ECM degradation and remodeling, directly influencing cancer progression. Moura et al. reviewed their role in chronic inflammation-induced colorectal cancer (CRC). Overexpression of MMP-1, -2, -3, and -7 promotes intestinal inflammation, IBD progression, and colitis-associated cancer (CAC), making them potential biomarkers of dysplasia and early neoplasia. Conversely, MMP-10 may exert protective effects, while MMP-9 shows context-dependent roles, acting both pro-inflammatory and anti-tumorigenic. These findings highlight the need to prospectively evaluate individual or combined MMPs as diagnostic and prognostic biomarkers.

Hu et al. examined heat shock protein 70 (HSP70), a chaperone implicated in multiple cancer-related processes. HSP70 regulates cell proliferation, apoptosis, ECM remodeling, and epithelial-to-mesenchymal transition (EMT). It also modulates immune responses, linking tumor biology with host defense. The review integrated evidence from clinical studies across diverse cancers, presenting HSP70 as a promising biomarker and potential therapeutic target.

The collection of articles in this Research Topic provides a comprehensive overview of the roles of the extracellular matrix (ECM) and immunity in cancer, highlighting how their interplay influences disease diagnostics, prognosis, and therapeutic strategies. The contributions introduce innovative methodologies and approaches that advance the identification of biomarkers capable of predicting responses to both conventional therapies and immunotherapies. Furthermore, the articles underscore the importance of deeper investigation into the tumor stroma and its individual components, offering novel perspectives for improving cancer diagnosis, understanding disease progression, and developing effective treatments.
